# A recognition of exosomes as regulators of epigenetic mechanisms in central nervous system diseases

**DOI:** 10.3389/fnmol.2024.1370449

**Published:** 2024-03-11

**Authors:** Shunxin Hu, Lei Feng, Zhonghong Yang, Xuechen Fan, Haozheng Gao, Tiancai Yang

**Affiliations:** ^1^Shandong First Medical University, Tai'an, China; ^2^Jining First People's Hospital, Jining, China

**Keywords:** exosomes, miRNA, lncRNA, methyltransferase, brain

## Abstract

Exosomes, vesicular structures originating from cells, participate in the conveyance of proteins and nucleic acids. Presently, the centrality of epigenetic modifications in neurological disorders is widely acknowledged. Exosomes exert influence over various epigenetic phenomena, thereby modulating post-transcriptional regulatory processes contingent upon their constituent makeup. Consequently, the heightened attention directed toward exosomes as instigators of epigenetic alterations has burgeoned in recent years. Notably, exosomes serve as vehicles for delivering methyltransferases to recipient cells. More significantly, non-coding RNAs, particularly microRNAs (miRNAs), represent pivotal contents within exosomes, wielding the capacity to influence the expression of diverse factors within the cerebral milieu. The transfer of these exosomal contents amidst brain cells, encompassing neuronal cells and microglia, assumes a critical role in the genesis and progression of neurological disorders, also, this role is not limited to neurological disorders, it may deal with any human disease, such as cancer, and cardiovascular diseases. This review will concentrate on elucidating the regulation of exosome-induced epigenetic events and its subsequent ramifications for neurological diseases. A more profound comprehension of the involvement of exosome-mediated epigenetic regulation in neurological disorders contributes to a heightened awareness of the etiology and advancement of cerebral afflictions.

## 1 Introduction

As is commonly acknowledged, the intricate structure of the brain, functioning as the “supreme command” in human beings, assumes an indispensable role in the processes of growth and development. Consequently, the onset of central nervous system diseases (CNS) is invariably accompanied by intricate and varied pathophysiological mechanisms. CNS encompasses a spectrum of maladies, spanning neurodegenerative diseases, cerebrovascular diseases, brain tumors, traumatic brain injuries, and intracranial infections. Manifestations of these disorders may range from mild impairments in motor, speech, sensory, and cognitive functions to more severe outcomes such as coma, and in extreme cases, deep coma and brain death (Naval et al., 2011). Presently, approximately 1 in 6 individuals worldwide grapple with CNS disorders, imposing a substantial burden on individuals, families, and society at large (Zhou et al., 2021). Despite the advancements in contemporary medical science, the diagnosis and treatment of CNS disorders confront formidable challenges, with the presence of the blood–brain barrier (BBB) standing out as a primary impediment (Piguet et al., 2021). In physiological states, the BBB offers protective fortification to the brain; however, it concurrently impedes the transit of the majority of therapeutic agents into the cerebral domain (Piguet et al., 2021). The emergence of exosomes has instilled optimism among scholars, as they hold promise for traversing the blood–brain barrier and gaining access to the brain. Exosomes are nanoscale membrane-bound vesicles and exhibit natural blood–brain barrier (BBB) traversing ability, which enables their application as drug delivery vehicles for brain disease treatment (Rehman et al., 2023). Immune exosomes loading self-assembled nanomicelles can traverse the blood–brain barrier for effective prevention of glioma recurrence (Cui et al., 2023). In recent years, artificially engineered exosomes have been developed as better alternatives to natural exosomes in terms of large-scale production, standardized isolation, drug encapsulation, stability and quality assurance. Manufactured exosomes are considered as potentially effective carriers for chemical and biological therapeutics, as we can control circulation time and selectivity (Abdelsalam et al., 2023).

Exosomes, measuring approximately 40–100 nm in size, constitute nanoscale extracellular lipid bilayer vesicles secreted by nearly all cell types under both physiological and pathological conditions (Kalluri and LeBleu, 2020). The story of the origins of exosome research arguably begins with the Chargaff’s studies of coagulation (Chargaff, 1945), then, Peter Wolf described a “material in minute particulate form, sedimentable by high-speed centrifugation and originating from platelets, but distinguishable from intact platelets” which he described as ‘platelet dust’ (Wolf, 1967). Subsequently, Nunez et al., described the presence of small (1–10 nm) extracellular vesicles in the bat thyroid gland during arousal from hibernation. We do believe that this paper was one of the first to describe the presence of multivesicular bodies (MVBs) close to the apical membrane (Nunez et al., 1974). Until 1983, two seminal and complementary papers published by the Johnstone and Stahl laboratories made a watertight case for the release of intraluminal vesicles from the cell, and defined them as exosomes (Harding et al., 1983; Pan and Johnstone, 1983). Initially perceived as a mechanism for the disposal of cellular debris, research advancements have unveiled the pivotal role of exosomes in facilitating cell-to-cell communication and maintaining cellular homeostasis through the transfer of nucleic acids, specific proteins, and lipids between cells (Liu et al., 2021). This regulatory function is, however, a double-edged sword, as exosomes possess the capability to modulate cellular properties. Present ubiquitously in the body, exosomes derived from distinct cell types exhibit variations in size and content, actively participating in diverse physiological and pathological processes such as immune response (Noonin and Thongboonkerd, 2021), cardiovascular and cerebrovascular diseases (Han et al., 2022), cancer initiation and progression (Mashouri et al., 2019), neuronal information transmission, and central nervous system diseases (Frühbeis et al., 2020). The inherent capacity of exosomes to regulate intricate intracellular pathways enhances their potential for therapeutic intervention across a spectrum of diseases, including neurodegenerative conditions and cancer.

Epigenetics, the study of regulatory mechanisms governing gene expression and gene interactions, addresses the fundamental issue of core regulation in the transmission of genetic information from the genome to the transcriptome (Jeffries, 2020). Epigenetic modification, a complex and reversible heritable process altering gene function, emerges as a central theme in basic neuroscience research, demonstrating its paramount role in the intricacies of nervous system structure and function. Epigenetic factors orchestrate various aspects of brain development, neurogenesis, synaptic plasticity, stress response, aging, and even the intergenerational inheritance of cognitive and behavioral phenotypes (Qureshi and Mehler, 2018). Translational research underscores the contribution of dysregulated epigenetic mechanisms to the onset of neurological diseases (Surguchov et al., 2017). Translational research underscores the contribution of dysregulated epigenetic mechanisms to the onset of neurological diseases. Targeting these processes in disease models holds the potential to substantially mitigate pathological changes and alleviate symptoms, encompassing the relief of neurodegeneration, promotion of nerve regeneration, and restoration of cognitive function (Gräff et al., 2012). The characterization of epigenetic and genome-wide epigenomic profiles, along with the regulation of epigenetic factors, introduces a novel and potent paradigm for identifying, monitoring, preventing, and potentially reversing neurological diseases.

### 1.1 Biogenesis and secretion of exosomes

**Figure 1:**
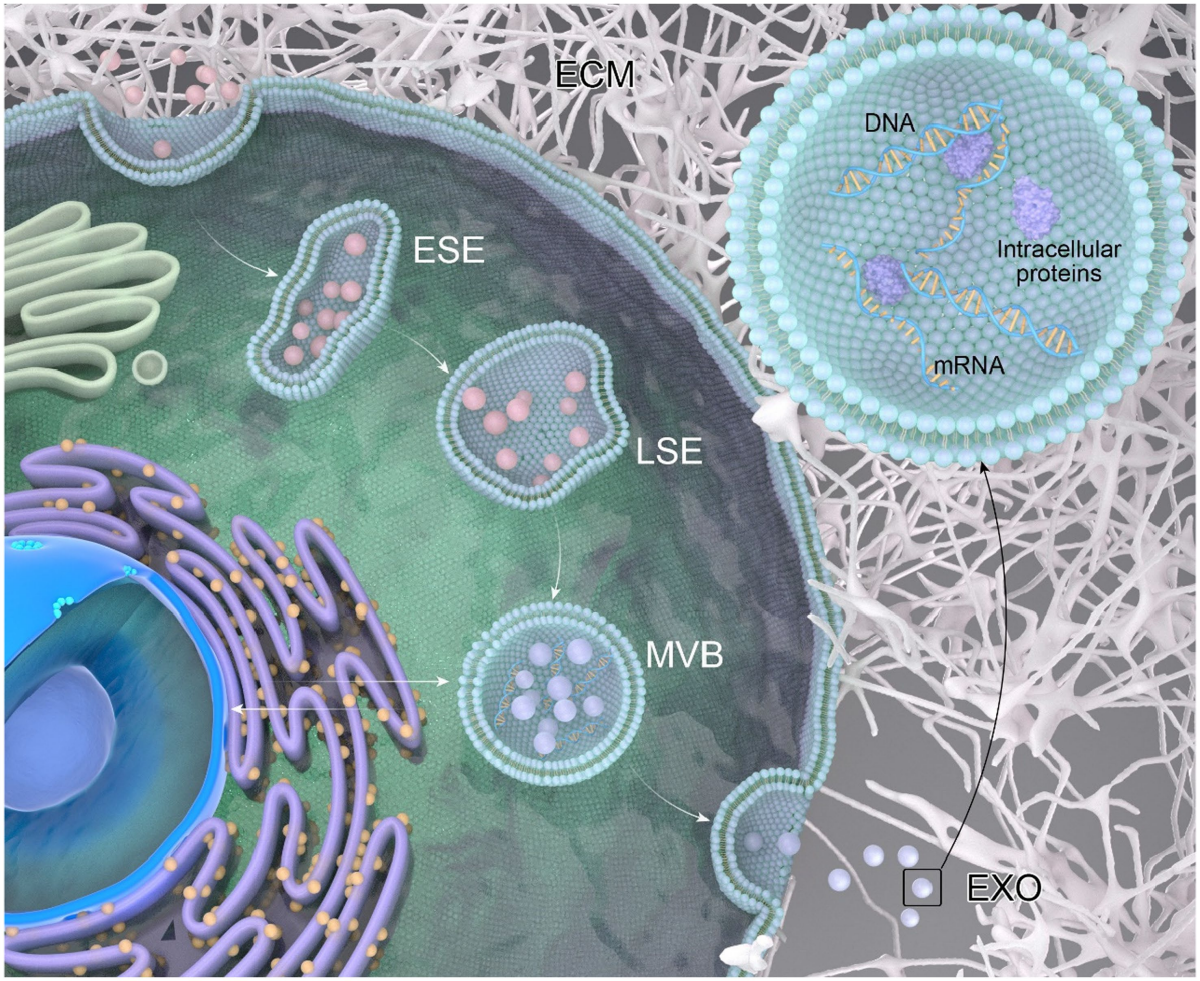
The process of exosome generation. Extracellular components can enter the cell through endocytosis and by invagination of the cell membrane. The vesicles formed during this process can fuse with selective early endosomes (SEEs), then transform into selective late endosomes (SLEs). The second invagination of the SLEs leads to the formation of intraluminal vesicles (ILVs). The ILVs then transform into multivesicular bodies (MVBs), which can fuse with lysosomes or autophagosomes for degradation, or fuse with the plasma membrane to release the ILVs as exosomes. A multivesicular body (MVB), generated from an endocytic cisterna by the accumulation of vesicles, exhibits small membrane curvatures corresponding to distinct microdomains.

The process of exosome generation (Figure 1) is a continuous and intricate sequence involving the double invagination of the plasma membrane and the formation of intracellular multivesicular bodies (MVBs) (Kalluri and LeBleu, 2020). The biogenesis initiates with the *de novo* formation of early sorting endosomes (ESEs). Initially, the plasma membrane undergoes invagination, forming a cup-shaped structure that encapsulates cell surface proteins and extracellular components, including soluble proteins, lipids, metabolites, small molecules, and ions (Mathieu et al., 2019). ESEs, post-formation, undergo fusion with the endoplasmic reticulum (ER), the trans-Golgi network (TGN), or pre-existing ESEs. Subsequently, ESE matures into late sorting endosomes (LSE) (Sahoo et al., 2021). The second invagination within LSE results in the formation of intraluminal vesicles (ILVs), facilitating the selective loading of future exosomes and permitting the entry of cytoplasmic components into the newly formed ILVs. LSE further matures into multivesicular bodies (MVBs), which can undergo degradation through fusion with lysosomes or autophagosomes, or release ILVs by fusing with the plasma membrane, transforming them into exosomes (Colombo et al., 2014).

Cells release exosomes in response to specific stimuli or as part of normal physiological processes (Kourembanas, 2015). These released exosomes function as carriers of molecular information, transferring cargo molecules from parent to recipient cells, thereby regulating cell-to-cell communication involved in various physiological and pathological processes. The quantity and composition of exosomes reflect the state of the originating cell. Whether under pathological or physiological conditions, the contents of exosomes are meticulously regulated by parent cells, transmitting specific information to other cells for specialized functions (Kilchert et al., 2016). Conversely, the functional status of parent cells can be inferred by analyzing their exosome contents, transmitting signals and molecules through the intercellular vesicle communication pathway, thereby exerting local or systemic effects (Isaac et al., 2021).

Exosomes, with diverse biological functions spanning multiple areas of biology, are the focus of this review specifically concerning diseases of the central nervous system. Within the context of the central nervous system, exosomes released by neurons, glial cells, and other cells form a complex network of interconnected information, influencing information transmission, physiology, and pathological effects within the system (Li et al., 2023).

### 1.2 Epigenetic regulation

The transmission of phenotype from one generation to the next is a fundamental aspect of life continuity in multicellular organisms, primarily achieved through mitosis, involving the replication and transmission of the organism’s genomic DNA. However, alongside genomic inheritance, the transmission of cell fate from parent to offspring also relies on the inheritance of epigenetic information. Mitotic inheritance necessitates the survival of epigenetic information despite a two-fold dilution of cell contents with each cell division. In each generation of organisms, all distinct cell types in the human body are re-established, implying that the majority of cell state information appears to undergo erasure or reprogramming in the germline of multicellular organisms (Takahashi et al., 2023).

Genetic and molecular investigations into mitosis and transgenerational epigenetic patterns have unveiled several key pathways involved in epigenetic gene regulation: (a) transcription factors (Roy et al., 2021), (b) chromatin structure (Stewart-Morgan et al., 2020), (c) covalent DNA modifications (Roy et al., 2021), (d) small RNAs (Holoch and Moazed, 2015), (e) prions (Viré and Mead, 2023), and sequence-specific DNA-binding proteins, serving as the primary determinants of cell fate in multicellular organisms. The most widely recognized forms of epigenetic regulation include DNA methylation, histone modifications, and non-coding RNAs (ncRNAs) (Li, 2021). Notably, prion-mediated inheritance primarily constitutes an epigenetic model for fungi (Viré and Mead, 2023).

Exosomes, comprising various contents, house DNA methyltransferases (Shrivastava et al., 2021) and small RNAs (Li et al., 2022), both capable of influencing epigenetic modifications. However, the official identification of these crucial genetic materials in exosomes did not occur until 2007 (Valadi et al., 2007). Exosomes derived from diverse tissues and cells exhibit varying quantities and sizes of DNA methyltransferases and small RNAs, signifying their distinct roles in epigenetic regulation. Wu et al., found that miR-124-3p delivered by exosomes from heme oxygenase-1 modified bone marrow mesenchymal stem cells inhibits ferroptosis to attenuate ischemia–reperfusion injury in steatotic grafts (Wu et al., 2022). In addition, exosomes released from Bone-Marrow Stem Cells ameliorate hippocampal neuronal injury through transferring miR-455-3p (Gan and Ouyang, 2022) and miR-31 from adipose stem cell-derived extracellular vesicles promotes recovery of neurological function after ischemic stroke by inhibiting TRAF6 and IRF5 (Lv et al., 2021).

### 1.3 Exosomes regulate epigenetic modifications

Exosomes play a pivotal role in the effective regulation of various epigenetic modification processes, including DNA methylation, acetylation, phosphorylation, and the expression of regulatory non-coding RNAs, thereby influencing the onset and progression of diseases (Zhao et al., 2023). For instance, hUC-mesenchymal stem cell-derived exosomes (hUC-MSC-EXO) have been found to enhance the expression of miR-4553p in response to IL-6 stimulation. Analysis through Western blot and QRT-PCR demonstrated a significant reduction in the expression of PIK3r1 at both mRNA and protein levels in macrophages in the presence of miR-4553p. PI3K, a key factor in inhibiting IL-6-related signaling pathways, is suggested to be suppressed by miR-4553p, leading to the inhibition of macrophage activation by downregulating the target gene PIK3r1. Importantly, hUC-MSC-EXO mitigates the release of IL6 and other inflammatory factors by macrophages through the promotion of miR-4553p expression. This targeted inhibition of PIK3r1 curtails the overactivation of immune cells such as macrophages/monocytes, attenuates inflammation, and preserves systemic homeostasis (Shao et al., 2020).

Another example involves stem cell-derived exosomes, which elevate the expression of two miRNAs, miR-135b-5p and miR-499a-3p, promoting angiogenesis in endothelial cells under blue light stimulation (Yang et al., 2019). In the context of bladder cancer progression, exosomes from bladder cancer cells contain a long non-coding RNA, lymph node metastasis-associated transcript 2 (LNMAT2), loaded through interaction with heterogeneous ribonucleoprotein A2B1 (hnRNPA2B1). Subsequently, internalized exosomal LNMAT2 in human lymphoid endothelial cells upregulates prospero homology box 4 (PROX1) expression through epigenetic mechanisms, ultimately leading to lymphangiogenesis and lymphatic metastasis, thereby promoting bladder cancer progression (Tchurikov et al., 2015; Chen et al., 2019).

Moreover, an HIV-1 promoter targets zinc finger protein (ZFP-362) fused to the active domain of DNA methyltransferase 3A, inducing long-term stable epigenetic repression of HIV-1. Cells engineered to produce exosomes encoding the RNA package of this HIV-1 repressor protein exhibit epigenetic inhibition of viral replication, delaying or inhibiting disease progression (Shrivastava et al., 2021). Additionally, human milk exosomes (MEX) and their miRNAs can enter the systemic circulation, potentially affecting epigenetic processes in various organs, including the liver, thymus, brain, pancreatic islets, beige, brown and white adipose tissue, and bones (Melnik et al., 2021). Translational evidence indicates that MEX and its miRNAs control the expression of global cellular regulators, such as DNA methyltransferase 1, important for upregulating developmental genes, and receptor interaction protein 140, essential for the regulation of multiple nuclear receptors. MEX-derived miRNA-148a and miRNA-30b may stimulate the expression of uncoupling protein 1, a key thermogenic inducer converting white adipose tissue to beige/brown adipose tissue (Shore et al., 2010).

Furthermore, exosome-delivered long non-coding RNA (lncRNA) UFC1 has been implicated in promoting non-small cell lung cancer progression through EZH2-mediated epigenetic silencing of PTEN expression. Mechanistically, UFC1 binds to EZH2, facilitating its accumulation in the PTEN gene promoter region, resulting in trimethylation of H3K27 and inhibition of PTEN expression. Exosome-borne UFC1, derived from non-small cell lung cancer cells, promotes the proliferation, migration, and invasion of these cells through UFC1-mediated metastasis (Zang et al., 2020). In summary, exosomes exhibit the capacity to either promote or inhibit disease progression by upregulating or silencing associated genes through intricate epigenetic mechanisms.

## 2 Epigenetic contents of exosomes for central nervous system diseases

### 2.1 DNA methyltransferases in the central nervous system (CNS)

DNA methylation involves the transfer of a methyl group to the fifth carbon of a DNA cytosine residue, resulting in the formation of a specific methylation structure (5-MC). This process predominantly occurs on the CPG island of the gene promoter region, leading to transcriptional silencing. The catalysis of this process is facilitated by the DNA methyltransferase family (DNMT), where different members play distinct roles in DNA methylation (Shrivastava et al., 2021). Aberrant DNA methylation is strongly implicated in various diseases; for instance, aberrant hypermethylation is a crucial epigenetic modification mechanism in atherosclerosis (Li et al., 2022). Overexpression of DNMT1 and DNMT3A contributes to abnormal DNA methylation of tumor suppressor genes (TSGs), consequently promoting pituitary adenoma invasion. Therefore, DNA methylation is closely linked to the onset and progression of aggressive pituitary adenomas (Kilchert et al., 2016). DNA methylation-based biomarkers and epigenetic therapies play pivotal roles in the early diagnosis and prognosis of various diseases. DNA methylation serves as a diagnostic marker for conditions such as vitamin deficiencies, neurodegenerative diseases (Martínez-Iglesias et al., 2023), meningioma (Choudhury et al., 2022), cerebrovascular diseases (Martínez-Iglesias et al., 2020), neuroinflammation (Dai et al., 2021), and psychiatric disorders (Feng and Fan, 2009).

### 2.2 Brain tumors

Currently, there is significant interest among researchers regarding the role of DNA methylation in exosomes in neurotumors. Epigenetic alterations have become a prominent feature of molecular pathology in the primary category of brain diseases (Qureshi and Mehler, 2013). Glioma, a complex and heterogeneous tumor, comprises not only tumor cells but also various non-tumor cell types, including astrocytes, microglia, endothelial cells, and immune cells, collectively constituting a complex glioma microenvironment (Quail and Joyce, 2017). Emerging evidence suggests that communication between tumor cells and components in the glioma microenvironment can directly influence various hallmark features of glioma (Godlewski et al., 2015). Elevated levels of extracellular vesicles (EVs) have been observed in the circulation of patients with glioblastoma and other cancer types, indicating that circulating tumor-derived EVs may serve as valuable biomarkers for monitoring treatment response and aiding in tumor diagnosis (Osti et al., 2019; Ricklefs et al., 2019).

The presence of O-6-methylguanine-DNA methyltransferase (MGMT) significantly impacts temozolomide (TMZ) therapy (Oldrini et al., 2020). Epigenetic silencing, achieved through promoter methylation of the MGMT gene, hinders the synthesis of this enzyme and stands as the sole known biomarker for TMZ response (Jha et al., 2010). Genomic rearrangements of MGMT help alleviate TMZ resistance both *in vitro* and *in vivo*, and these rearrangements can be detected in tumor-derived exosomes (Oldrini et al., 2020). Simultaneously, tumor-derived exosomes exhibit the ability to carry TMZ and dihydrotanshinone (DHT), contributing to the reversal of drug resistance and enhancing lesion-targeted drug delivery (Wang et al., 2022) for the targeted therapy of glioma.

### 2.3 Neurodegenerative diseases

Neurodegenerative diseases, encompassing Parkinson’s disease (PD), Alzheimer’s disease (AD), amyotrophic lateral sclerosis (ALS), among others (Hou et al., 2019), pose a significant health challenge. Age is the most prominent risk factor for these diseases, and cognitive decline is inherently associated with aging. Consequently, chromatin alterations occurring during brain aging emerge as crucial targets for preventing cognitive deterioration, with DNA methylation playing a pivotal role in this context (Younesian et al., 2022). The differential CpG-2 methylation status of α-synuclein (SNCA) in leukocytes in the blood of PD patients may serve as a novel diagnostic indicator for PD (Tan et al., 2014). However, existing research on the role of DNA methylation in neurodegenerative diseases primarily focuses on diagnostic markers, necessitating additional efforts to delve into deeper mechanisms and potential treatments.

### 2.4 Other neurological disorders

While DNA methylation is prevalent in various neurological disorders, limited research has been conducted in this realm. Gulf War Illness (GWI), a chronic multisymptomatic disease with central nervous system damage as a commonly reported symptom, including memory dysfunction and depression, remains challenging to diagnose and lacks effective treatments for poorly understood reasons. In the context of GWI, alterations in DNA methylation and hydroxymethylation levels in exosomes have been observed (Pierce et al., 2016), indicating the involvement of epigenetic changes in exosomes in GWI and offering new perspectives for future diagnosis and treatment. Additionally, in stroke patients, exosomes released by curcumin-treated cells have been found to inhibit DNA methylation levels, thereby mitigating the harm caused by stroke (Liu et al., 2009).

## 3 Small RNA in the central nervous system (CNS)

RNA modifications constitute a crucial aspect of epigenetic modifications (Melnik et al., 2021). Comparable to DNA modifications, cellular RNA undergoes various chemical modifications, such as N6-methyladenosine (m6A) for mRNA. Among these modifications, m6A stands out as the most abundant epigenetic modification of RNA, primarily catalyzed by a methyltransferase complex comprising METTL3, METTL4, and other protein subunits. Aberrant m6A modification can result in transcriptional abnormalities, leading to irregular translation procedures that foster tumorigenesis and progression. Studies indicate that m6A modification also plays a significant role in the onset and progression of neurological diseases by regulating immune cells and RNA. The YTH domain family 2 (YTHDF2), an m6A-binding protein, has been identified, and knockdown of YTHDF2 has been linked to increased inflammation. YTHDF2 expression strongly correlates with the development of inflammatory bowel disease (IBD) (Shore et al., 2010; Hock et al., 2017). The chemical modification of these RNAs is pivotal in RNA metabolism. Exosomes deliver molecules through a variety of pathways, manipulate the epigenetics of cells, and influence disease progression (Table 1).

**Table 1:** The biological functions of exosomal ncRNAs in CNS.

Exosomal cargo	Non-coding RNA type	Brain disorders	Effect	Reference
miR-29c	miRNA	AD	Affecting amyloid beta generation	Chen et al. (2019)
miR-339-5p	miRNA	AD	Down-regulates protein expression of β-site amyloid precursor protein-cleaving enzyme 1	Long et al. (2014)
miR-4813-3p	miRNA	PD	Mediating inability to repress autophagic program	Ma et al. (2023)
miR-34a-5p	miRNA	PD	Regulating aspects of neurogenesis and synaptogenesis	Grossi et al. (2020)
circEPS15	circRNA	PD	Maintain mitochondrial homeostasis	Zhou et al. (2023)
miR-149	miRNA	PA	Suppressive effects on tumor growth and angiogenesis	Chang et al. (2023)
miR-760-3p	miRNA	CI/RI	Inhibiting neuron ferroptosis	Wang et al. (2023)
circ_0072083	circRNA	Glioma	Enhances temozolomide resistance in glioma	Ding et al. (2021)
miR-146a-5p	miRNA	Ischemic stroke	Reduces microglial-mediated neuroinflammation	Zhang et al. (2021)
miR-182-5p	miRNA	Ischemic stroke	Inhibit neuroinflammation after stroke	Ding et al. (2023)
miR-17-5p	miRNA	Ischemic stroke	Inhibiting cell senescence, endothelial cell oxidative stress, apoptosis, and dysfunction	Pan et al. (2023)
miR-2861	miRNA	SCI	Promote blood-spinal cord barrier repair and motor function recovery	Kong et al. (2023)

AD, Alzheimer’s disease; PD, Parkinson disease; PA, pituitary adenomas; CI/RI, cerebral ischemia–reperfusion injury; SCI, spinal cord injury.

### 3.1 Neurodegenerative diseases

The role and mechanisms of small RNAs in neurodegenerative diseases have garnered significant attention from researchers. Small RNAs serve not only as diagnostic markers for neurodegenerative diseases but also play a substantial role in disease occurrence and development. Various scientific evidence suggests the existence of global and gene-specific epigenetic changes at both peripheral and brain levels in patients with neurodegenerative diseases such as Alzheimer’s disease (AD) (Coppedè, 2020), Parkinson’s disease (PD) (Zhang et al., 2023), and amyotrophic lateral sclerosis (ALS) (Coppedè, 2020).

For instance, in patients with PD and AD, substantial amounts of miRNA are present in cerebrospinal fluid and blood exosomes, exhibiting differences from normal levels (Gui et al., 2015). In amyotrophic lateral sclerosis (ALS), RNA within extracellular vesicles released by muscles can disrupt motor neurons (MNs), denaturing and promoting disease progression (Le Gall et al., 2022). Additionally, the Drosophila brain can acquire circ_sxc by ingesting adipose tissue exosomes that traverse the blood–brain barrier. circ_sxc inhibits the expression of miR-87-3p in the brain, regulating the expression of neuroreceptor ligand proteins (5-HT1B, GABA-B-R1, Rdl, Rh7, qvr, NaCP60E), ensuring the normal function of synaptic signal transduction in brain neurons. However, with age, this regulatory mechanism is dysregulated due to the downregulation of fat exosomal circ_sxc, accelerating “aging” in the brain and potentially hastening the development of neurodegenerative diseases (Li et al., 2023).

### 3.2 Brain tumors

The presence of brain tumors, particularly gliomas, remains a significant challenge for neuroscientists due to their rapid growth, high recurrence rate, and malignant nature. In glioblastoma multiforme (GBM), the expression levels of one small non-coding RNA (RNU6-1) and two microRNAs (miR-320 and miR-574-3p) are significantly associated with GBM diagnosis, with RNU6-1 potentially serving as an independent predictor for GBM diagnosis (Manterola et al., 2014). Low serum levels of miR-485-3p predict reduced survival in glioblastoma patients (Wang et al., 2017).

In terms of treatment, blood exosomes (Exos) have been chosen as the delivery vehicle, combining cytoplasmic phospholipase A2 (cPLA2) knockdown and metformin for GBM treatment. This approach can effectively traverse the blood–brain barrier, reaching the brain and GBM tissues. It inhibits the mitochondrial energy metabolism of GBM, thereby suppressing tumor growth and extending survival (Zhan et al., 2022). Tumors can also influence the tumor microenvironment (TME) by secreting exosomal microRNAs (miRNAs). Exosomal miRNAs inhibiting tumors are absorbed by immune cells in TME and converted into cancer promoters, thus impeding tumor cell proliferation and delaying disease progression (Qi et al., 2022). Considering the effective penetration of exosomes through the blood–brain barrier and the significant advancements in tumor treatment at the genetic level, the prospects for glioma treatment through exosome drug delivery and the study of RNA in exosomes and their cargo hold promising potential for major breakthroughs.

### 3.3 Cerebrovascular diseases

Cerebrovascular diseases exert a substantial impact on global human health, posing a significant threat to physical well-being and contributing to a considerable economic burden worldwide. They stand as the primary cause of disability (Gutierrez and Esenwa, 2015), and research on exosomes for cerebrovascular diseases is gaining traction. Notably, exosomal let-7b-5p miRNAs may serve as a potential prognostic marker for poor outcomes after stroke. Exosomal miR-223-3p derived from mesenchymal stem cells can facilitate the transformation of M1 microglia into M2 microglia, mitigating cerebral ischemia/reperfusion injury (Zhao et al., 2020). Exosomes from M2 phenotypic microglia can target Notch1 to ameliorate neuronal death induced by ischemia–reperfusion injury (Zhang et al., 2021). M2 microglia-derived exosomes attenuate ischemic brain injury and promote neuronal survival through exosomal miR-124 (Song et al., 2019). In patients with cerebral infarction, miRNA-modified exosome therapy has demonstrated improvements in infarct volume and neurobehavior (Yu et al., 2022). Moreover, in the hypoxic state following a stroke, RNA within exosomes secreted by neuronal cells can reduce neuronal activity, inhibit axon and dendrite growth, and expedite stroke progression (Chiang et al., 2021).

### 3.4 Mental disorders

Mental disorders encompass a diverse range of conditions, with depression emerging as one of the most challenging. Depression is characterized by significant and persistent low mood, sluggish thinking, and reduced motivation, severely impeding psychosocial functioning and diminishing overall quality of life. In 2008, the World Health Organization ranked depression as the third leading contributor to the global burden of disease, projecting it to ascend to the foremost position by 2031 (Malhi and Mann, 2018). While comprehensive models or theories for depression research are currently lacking, substantial progress has been made recently in understanding the interplay between exosomes, their contents, and depression.

Dysregulation of numerous microRNAs (miRNAs), including miR-16, miR-451, miR-223, and miR-182, has been suggested to be associated with depressive conditions in diverse patients, offering potential markers for depression (Gururajan et al., 2016). Studies indicate that neurons release exosomes containing miR-9-5p. Elevated serum levels of miR-9-5p in individuals with depression stimulate the polarization of M1 microglia, triggering excessive release of pro-inflammatory cytokines such as interleukin-1β (IL-1β), interleukin-6 (IL-6), and tumor necrosis factor-α (TNF-α), thereby exacerbating neurological damage (Xian et al., 2022). Furthermore, microglia transfer microRNA miR-146a-5p via exosome secretion to inhibit neurogenesis in depression. Overexpression of miR-146a-5p in the dentate gyrus of the hippocampus suppresses spontaneous electrical emission of neurogenesis and excitatory neurons by directly targeting Krüppel-like factor 4 (KLF4). Downregulation of miR-146a-5p expression ameliorates neurogenesis deficits in the DG regions and depressive-like behavior in rats. Simultaneously, circular RNA ANKS1B mediates post-transcriptional regulation of KLF4 expression as a miRNA chelator of miR-146a-5p. Thus, miR-146a-5p plays a pivotal role in regulating neurogenesis in the pathological processes associated with depression (Fan et al., 2022).

## 4 Conclusion and future prospects

Neurological diseases have afflicted humanity for numerous years, and though some efforts have been made to alleviate suffering, there is still a considerable journey ahead in their treatment. The presence of exosomes, well-established as cell-to-cell communication mediators (Fyfe et al., 2023), has been confirmed for many years. Exosomes can function as intercellular communication molecules. These vesicles often transport proteins and nucleic acids between cells, potentially contributing to the pathogenesis of various neurological diseases. This article provides evidence for exosome-mediated epigenetic mechanisms that regulate factor transfer, emphasizing the profound impact of exosome-transported methyltransferases on gene expression in recipient cells. While exosomes transport methyltransferases, they may also influence the expression of these enzymes in recipient cells indirectly. Moreover, exosomes transfer various non-coding RNAs in the brain microenvironment, including miRNAs, lncRNAs, and circRNAs, among others. Despite the ongoing emergence of relevant information, a complete chain of evidence is still lacking in many published reports, particularly regarding the detailed mechanisms of up-or downregulation of factors/non-coding RNAs that directly interfere with the action of development of central nervous system diseases. We only described two types of epigenetic mechanisms related to exosomes in this review, small RNA and DNA methyltransferases, this is limited. There are many types of RNA, and further research is needed to investigate whether RNA such as tRNA and siRNA play a deeper role in central nervous system diseases.

While conclusive knowledge is currently elusive, there is substantial evidence supporting the role of exosome-mediated regulation of epigenetic mechanisms in neurons and surrounding cells, contributing to the onset and progression of diseases. Conducting focused studies on these mechanisms will be crucial for realizing the clinical potential of exosomes in neurological disease research and treatment. The identification or synthesis of novel chemicals capable of modulating exosome synthesis or function, along with the development of non-toxic approaches for utilizing exosomes as therapeutic agents, holds the potential to rapidly advance this field. Further research is imperative to comprehensively understand the role of exosomes in epigenetic resistance, enabling the identification and validation of valuable targets for diagnosis and treatment of central nervous system diseases. Despite that there are few studies, we believe that epigenetic regulation of exosomes has great potential in the treatment of sleep disorders, anxiety and other diseases.

In fact, there have been two main technical hindrances that restrict the basic and applied research of exosomes. The first is how to simplify the exosome extraction procedure and improve the yield of exosomes; the second is how to effectively distinguish exosomes from other extracellular vesicles, especially from functional microvesicles (Fais et al., 2016). Various exosome separation strategies and devices have been suggested to facilitate the investigation of exosomes and their related biological functions. Through studying the nature of particular samples and specific application settings, we believe careful selection of isolation techniques (or a combination of isolation techniques) will help investigators address many of the challenges faced in current exosome studies (Yang et al., 2020). There are many methods available on the market that are currently being used in labs worldwide to obtain exosomes. Until now, six classes of exosome separation strategies have been reported, including ultra-speed centrifugation, ultrafiltration, immunoaffinity capture, charge neutralization-based polymer precipitation, size-exclusion chromatograph, and microfluidic techniques, with unique sets of advantages and disadvantages for each technique from different manufacturers (Supplementary Table S1). It is evident that currently available technology (as well as that of the future) will allow for the improvement of isolation methods and pave the way for novel findings in the years to come.

## Author contributions

SH: Writing – original draft. LF: Writing – review & editing. ZY: Writing – review & editing. XF: Writing – review & editing. HG: Writing – review & editing. TY: Writing – review & editing.
