# Supplementary material for: A recognition of exosomes as regulators of epigenetic mechanisms in central nervous system diseases
Source: Front Mol Neurosci. 2024 Mar 11;17:1370449. doi: 10.3389/fnmol.2024.1370449 (PMC10962328; doi:10.3389/fnmol.2024.1370449)
Supplement: Supplementary file 1 [file Table_1.docx]

Supplementary information

Table S1

| Isolation technique | Recovery rate | Specificity | Advantages | Disadvantages | Merchants | Reference |
| --- | --- | --- | --- | --- | --- | --- |
| Density gradient centrifugation | Low | High | 1.High properties and purity of products ;  2.Allowing separation of subpopulation of exosomes | 1.Cumbersome preliminary work, complicated opera-  tion, time-consuming;  2.Treatment capacity is limited by the load zone;  3.Hard to remove high-density chemi-  Cals;  4.subcellular water loss caused by hypertonic  reagents;  5.Not suitable for small volume diagnosis  6.Low portability | Sigma Aldrich | (Jeppesen et al., 2014) |
| Sequential ultracentrifugation | High | Low | 1.Simplified operation;  2.Simplified sample pretreatment  3. Low contamination risk with extra isolation reagents; 4.Suitable for large volume preparation | 1.Low RNA yield and mix with other kind of EVs like impurity proteins;  2. Potential mechanical damage due to high speed centrifugation | Abcam | (Doyle & Wang, 2019) |
| Size-exclusion chromatography | Middle | Middle | 1.High purity and sensitivity;  2.Not affected by  the high viscosity of the sample;  3.Prevents exosomes aggregation;  4.Easy to distinguish from high-density lipopro-  tein | 1.Relatively high device costs ;  2.Additional method for exosome enrichment is required | Sigma Aldrich | (Gámez-Valero et al., 2016; Konoshenko et al., 2018) |
| Immunoaffinity capture | Low | High | 1.Easy to use ;  2.Using ordinary equipment ;  3.Suitable for both small and large sample volume;  4.High efficiency | 1.Contaminants of protein aggregates, other extracellular vesicles and polymeric contaminants ;  2. Require complicated clean-up steps;  3.Affecting downstream analysis and quantification | Thermofisher;  Yesen | (Liu & Su, 2019) |
| Microfluidic-based isolation technique | Low | High | 1.Easy to automate and integrate;  2.High portability and purity ang low reagents;  3.Exosomes extrac-  tion and analysis can be combined | Low sample capacity | LabSpinner;  System Biosciences | (Momen-Heravi et al., 2012) |
| Ultrafiltration | Middle | Middle | 1. Fast procedure;  2.Low equipment cost | 1.Hard to remove soluble proteins;  2.Poor sustainability,  3.The external force may damage biological activity of  exosome[ | Cytiva pall | (Cheruvanky et al., 2007) |
| Polymer Precipitation | Low | High | 1.Using ordinary equipment  2.Suitable for both small and large sample volume  3.High efficiency | 1.Affecting downstream analysis and quantification;  2.Require complicated clean-up steps | Biosharp | (Soares Martins et al., 2018) |

References

Cheruvanky, A., Zhou, H., Pisitkun, T., Kopp, J. B., Knepper, M. A., Yuen, P. S. T., & Star, R. A. (2007). Rapid isolation of urinary exosomal biomarkers using a nanomembrane ultrafiltration concentrator. *American Journal of Physiology-Renal Physiology*, *292*(5), F1657–F1661. https://doi.org/10.1152/ajprenal.00434.2006

Doyle, L., & Wang, M. (2019). Overview of Extracellular Vesicles, Their Origin, Composition, Purpose, and Methods for Exosome Isolation and Analysis. *Cells*, *8*(7), 727. https://doi.org/10.3390/cells8070727

Gámez-Valero, A., Monguió-Tortajada, M., Carreras-Planella, L., Franquesa, M., Beyer, K., & Borràs, F. E. (2016). Size-Exclusion Chromatography-based isolation minimally alters Extracellular Vesicles’ characteristics compared to precipitating agents. *Scientific Reports*, *6*(1), 33641. https://doi.org/10.1038/srep33641

Jeppesen, D. K., Hvam, M. L., Primdahl‐Bengtson, B., Boysen, A. T., Whitehead, B., Dyrskjøt, L., Ørntoft, T. F., Howard, K. A., & Ostenfeld, M. S. (2014). Comparative analysis of discrete exosome fractions obtained by differential centrifugation. *Journal of Extracellular Vesicles*, *3*(1), 25011. https://doi.org/10.3402/jev.v3.25011

Konoshenko, M. Yu., Lekchnov, E. A., Vlassov, A. V., & Laktionov, P. P. (2018). Isolation of Extracellular Vesicles: General Methodologies and Latest Trends. *BioMed Research International*, *2018*, 1–27. https://doi.org/10.1155/2018/8545347

Liu, C., & Su, C. (2019). Design strategies and application progress of therapeutic exosomes. *Theranostics*, *9*(4), 1015–1028. https://doi.org/10.7150/thno.30853

Momen-Heravi, F., Balaj, L., Alian, S., Trachtenberg, A. J., Hochberg, F. H., Skog, J., & Kuo, W. P. (2012). Impact of Biofluid Viscosity on Size and Sedimentation Efficiency of the Isolated Microvesicles. *Frontiers in Physiology*, *3*. https://doi.org/10.3389/fphys.2012.00162

Soares Martins, T., Catita, J., Martins Rosa, I., A. B. Da Cruz E Silva, O., & Henriques, A. G. (2018). Exosome isolation from distinct biofluids using precipitation and column-based approaches. *PLOS ONE*, *13*(6), e0198820. https://doi.org/10.1371/journal.pone.0198820
